# Efficacy of swab-based RUT in detecting *H. pylori* infection: a systematic review and meta-analysis

**DOI:** 10.1186/s12876-025-04576-6

**Published:** 2026-03-27

**Authors:** Abdelaziz A. Awad, Mohamed A. Aldemerdash, Basma M. El-Khalifa, Ahmed Emara, Ali M. Othman, Manar A. Balouz, Youssef Narouz, Doaa A. Elmarzouky, Amira A. Albawri, Yara M. Harash, Ahmed M. Zaghloul, Ahmed Bahnasy, Ahmed Gad

**Affiliations:** 1 Faculty of Medicine, Al-Azhar University, Cairo, Egypt; 2 Faculty of Medicine, Sohag University, Sohag, Egypt; 3 Department of Clinical Oncology and nuclear medicine, Faculty of Medicine, Mansoura University, Mansoura, Egypt; 4 Faculty of Medicine, Kafr Elsheikh University, Kafr Elsheikh, Egypt; 5 Faculty of Medicine, Menoufia University, Menoufia, Egypt; 6 Faculty of Medicine, Ain Shams University, Cairo, Egypt; 7 Faculty of General Surgery and Medicine, Zagazig University, Zagazig, Egypt; 8 Faculty of Medicine, University of Science and Technology, Hawban, Yemen; 9 Faculty of Medicine, Hama University, Hama, Syria; 10 Haematology and Oncology Department, Medicine, Mayo Clinic, Scottsdale, AZ USA; 11 Hennepin County Medical Centre, Minneapolis, USA; 12 New Damietta, Damietta Egypt

**Keywords:** Helicobacter pylori, Swab, Sweeping, Rapid urease test

## Abstract

**Introduction:**

Helicobacter pylori (*H. pylori*) is a gram-negative bacterium found in the gastric mucosa of humans. In this systematic review, our objective was to assess the diagnostic performance of the swab technique in detecting *H. pylori* infection.

**Methods:**

We followed the PRISMA guidelines and searched four databases for studies comparing the efficacy of swabs or sweeping techniques in detecting *H. pylori* infection. Outcomes were diagnostic accuracy measures, such as sensitivity and specificity.

**Results:**

Four studies with 658 patients were included. Compared to conventional diagnosis methods, overall sensitivity was 0.93, with a 95% CI (0.85; 0.97). A confusion matrix was used to summarize the previously mentioned results. Gastric swabs reported a high sensitivity of 93.7%, with a high number of true positives = 285. However, test specificity is moderate (74.1%).

**Conclusion:**

These results indicate that gastric swab-based RUT may be helpful as an initial diagnostic test, specifically if a biopsy is difficult. More studies with large sample sizes are needed to confirm these findings.

**Supplementary Information:**

The online version contains supplementary material available at 10.1186/s12876-025-04576-6.

## Introduction

Helicobacter pylori (*H. pylori*) is a gram-negative bacterium found in the gastric mucosa of humans and is among the most prevalent bacterial infections, affecting over 50% of the global population [1]. It is associated with the development of several gastric diseases, such as gastric cancer and ulcers, atrophic gastritis, and mucosa-associated lymphoid tissue lymphoma [2, 3]. Therefore, correctly diagnosing *H. pylori* infection is crucial as an initial measure to eliminate and reduce *H. pylori*-related conditions.

The most common diagnostic tests for *H. pylori* infection include the rapid urease test (RUT), serum or urinary antibody test, stool antigen test, and incubation [4]. The standard treatment options that are used are proton pump inhibitors combined with at least two antibiotics [5]. However, we cannot certainly identify eradication and should be followed by monitoring tests after eradication, such as the urea breath test (UBT), stool antigen tests, and histologic examination (if the endoscopic assessment is required) [6, 7].

RUT is the most commonly used method, and it is performed using biopsy tissue. The tissue sample must be obtained from a site where the bacteria are present, and enough colony-forming units (CFU) of *H. pylori* must be included in the sample to get accurate results [8]. Furthermore, the administration of antibiotics and proton-pump inhibitors (PPIs) can result in false-negative results due to decreased bacterial load [9]. Despite obtaining several biopsies that have been suggested to increase the sensitivity of the RUT, it raises the risk of mucosal injury and complications, including bleeding [10].

RUT detects *H. pylori* via the urease that the organism produces, which is an indirect way [8]. Therefore, it remains uncertain if a tissue biopsy is the most suitable approach, considering that *H. pylori* exists in the mucosal layer. Like nasopharyngeal swabs, many studies hypothesized using a swab technique, in which a larger area can yield more *H. pylori* and allow for a more precise and less invasive diagnosis of *H. pylori* compared to a tissue biopsy.

In this systematic review, we aimed to assess the diagnostic performance efficacy of swab-based RUT (S-RUT) compared to traditional tissue-based RUT (T-RUT) for *H. pylori* infection.

## Methods

In this systematic review and meta-analysis, we followed the Preferred Reporting Items for Systematic Reviews and Meta-analyses (PRISMA) guidelines [11] and the Cochrane Handbook for Systematic Reviews of Interventions [12]. This review was registered on PROSPERO (CRD420251252680).

### Literature search

We conducted an electronic search in four medical databases (PubMed, SCOPUS, Cochrane, and Web of Science) until January 25, 2025. The search terms included Sweeping, swab, Helicobacter pylori, and *H. pylori.* The search strategies are detailed in Table S1.

### Eligibility criteria

We included studies comparing the swab technique in detecting *H. pylori* infection to a biopsy or urea breath test (UBT). Studies reporting diagnostic accuracy measures, such as sensitivity, specificity, and area under the curve (AUC), which refers to the area under the Receiver Operating Characteristic (ROC) curve, that measures a model’s ability to distinguish between classes.

Exclusion criteria comprised non-original studies, secondary research, studies with only abstracts or inaccessible full texts, non-extractable data or overlapping datasets, animal or in-vitro studies, duplicate studies, case reports, and non-English publications.

### Selection process

The literature was screened using Rayyan [13]. Two independent authors conducted the screening process while blinded to each other’s evaluations. A third author resolved any disagreements.

### Data extraction

A standardized Google spreadsheet was used to extract the data, including (1) Study characteristics and summary (Author, year of publication, country, age, gender, Gastric ulcer, gold standard test) and (2) diagnostic accuracy measures, such as sensitivity, specificity, and area under the curve (AUC). Any discrepancies between reviewers were resolved through discussion.

### Quality assessment

The QUADAS-2 instrument was used to assess the risk of bias. QUADAS-2 assesses four key domains: patient selection, index test, reference standard, and flow and timing. Each domain is thoroughly evaluated for risk of bias and applicability [14].

### Statistical analysis

Data synthesis was performed using R 4.5.2 and the mada package. Effect sizes were pooled as the logarithm of diagnostic odds ratios (DOR) using both a random-effects model [Der Simonian and Laird] and fixed fixed-effects model [Mantel-Haenszel] (with 95% confidence intervals, incorporating sensitivity (SEN), receiver operating characteristic (ROC), summary receiver operating characteristic (SROC), and specificity (SPE) measures [15].

## Results

### Literature search results

**Fig. 1:**
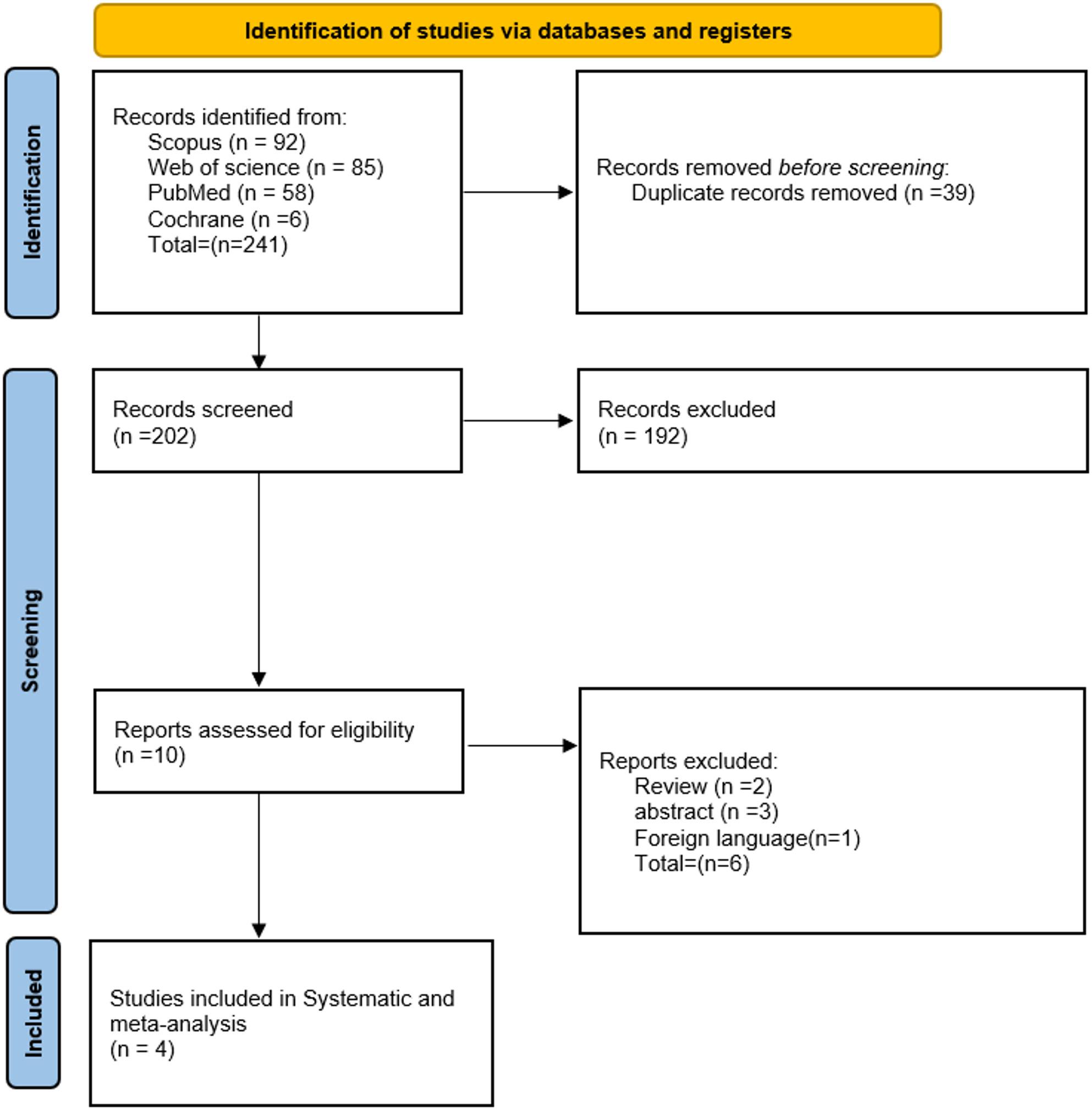
PRISMA flow diagram

241 studies were obtained from the literature search. Of them, 39 studies were identified as duplicates. After removing irrelevant abstracts, reviews, case reports, case series, non-English, and animal studies. 10 were eligible for full-text screening. Finally, four studies are included in this systematic review and meta-analysis [16–19]. Detailed literature search results are reported in the PRISMA flow diagram shown in Fig. 1.

### Characteristics of the included studies

We have included four studies in our review, three of which were observational [16, 17, 19], and only one was a double-blind, randomized controlled trial [18]. All the included studies compared the accuracy of gastric mucosal swabs in diagnosing *H. pylori* with gastric biopsy, except Yoshikawa et al. [17], where the gastric mucosal swab is compared to the Urea Breath Test (UBT). The summary of the included studies and baseline characteristics of the included populations are shown in Table 1.

**Table 1:** Characteristics of the included studies

Study ID	Study design	Country	Sample size, *n*	Study period	Age, mean (SD)	Gender, male, *n* (%)	Gastric ulcer, *n* (%)	Atrophic gastritis, *n* (%)	Gold standard test	Inclusion criteria	Swab Material and Size	Sweeping Procedure Details	Gastric Location Sampled	RUT Kit Used	Conclusion
Noh 2024 [16]	Prospective, crossover, single-center study	Korea	216	March 2019 and May 2022.	58.8 (11.0)	146 (67.6)	37 (17.1)	63 (29.2)	UBT, histology, and polymerase chain reaction	*H. Pylori* confirmed patients, who previously received eradication treatment and are scheduled for EGD.	Sterilized 6-mm circular pieces punched out from nonwoven fabric (LIVSEN SMMS BU3).	Swab grasped with forceps and swept back and forth 10 times. Gastric juices were not suctioned.	Side of the great curvature of the antrum and repeated for the corpus.	CLO kit (PYLO-PLUS),.	RUT combined with the sweeping method reported high sensitivity and accuracy, along with rapid detection of *H. Pylori* even after eradication. The technique was proven to be safe with no adverse events.
Yoshikawa 2024 [17]	A multicenter prospective observational study (prospective cross-sectional study)	Japan	94	March 2016 and December 2020	56.75 (12.51)	52 (57.3)	NA	57 (60.6)	Conventional gastric mucosa biopsy using forceps for RUT (Forceps RUT)	Patients underwent *H. pylori* infection assessment according to the Kyoto classification.	Nonwoven fabric (LIVSEN SMMS BU3) cut into pieces of 6 mm in diameter.	Sweeping continuously performed 10 times.	Antrum and corpus.	Pylo-Plus.	Results report high efficacy and safety of swab-RUT in detecting *H. Pylori* infection. It was also suitable for screening during EGD.
Soh 2023 [18]	Prospective cross-over study	Korea	69	July to December 2021	64.5(11.4)	34 (49.3)	2 (2.9)	41 (59.4)	Conventional tissue biopsy-based RUT	Adult patients (aged ≥ 19 years) are recommended EGD and *H. pylori* testing.	Cotton swab tip, separated and formed into an approximately 5 × 5 × 5 mm ball shape.	Sterile swab held with biopsy forceps and gently pressed against the wall while swabbing for 10 s along its circumference.	Antrum and corpus.	HP Kit, CKD Bio.	RUT reported high sensitivity and accuracy compared to the conventional method.
Noh 2020 [19]	Prospective, single-center study	Korea	279	November 2018 to July 2019.	59.76 (12.07)	192 (69.2)	39 (14)	63 (22.6)	Conventional biopsy sampling method in rapid urease test	Patients who underwent upper endoscopy and are recommended an *H. Pylori* test.	Small or large cotton (1-mm or 3-mm in original compressed form, expanding to around 5-mm or 7-mm).	One piece of cotton swabbed the anterior wall of the gastric antrum. A second piece swabbed the anterior wall of the upper corpus. The two pieces were analyzed in separate kits.	Anterior wall of the gastric antrum and anterior wall of the upper corpus.	Helicocheck^®^.	The sweeping methods reported high sensitivity and accuracy, along with rapid detection time. Also, it is potentially safe with no concern of mucosal damage and bleeding.

All quantitative data is measured as mean (standard deviation), while all qualitative data is reported as a number (percentage)

*Abbreviations*: *UBT* urea breath test; *H. Pylori* helicobacter pylori, *EGD* esophagogastroduodenoscopy, *RUT* rapid urease test

### Risk of bias assessment

Two studies were of low risk of bias [16, 19]. while the other two carried some concerns [17, 18]. A comprehensive summary of these evaluations is presented in Figure S1.

### Meta-analysis results

#### ROC plane and SROC curve results

**Fig. 2:**
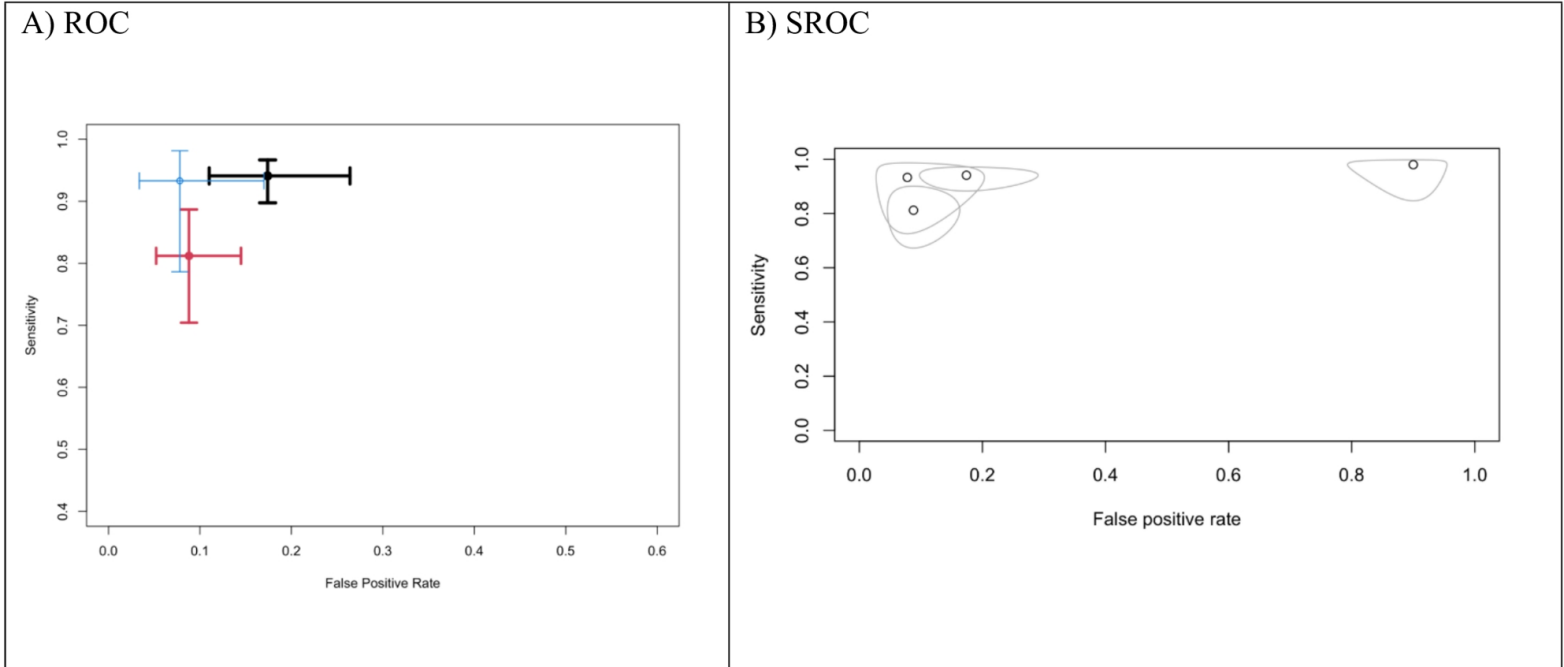
Forest plots for (**A**) ROC, (**B**) SROC

A receiver operating characteristic (ROC) plane was used to check the sensitivity and specificity of gastric swabs. According to the reported results, high sensitivity is reported in all the included studies, with variability in the specificity results indicating a high number of false positives; this can be attributed to the smaller sample size provided by Soh et al. [18] Shown in (Fig. 2A).

A summary receiver operating characteristic (SROC) curve was constructed to account for the heterogeneity between the included studies. The results were consistent with what was previously mentioned. The higher sensitivity of gastric swabs was consistent across all included studies; however, moderate to high specificity was reported due to variability between the included populations, as shown in Fig. 2B.

#### Diagnostic odds ratio

**Fig. 3:**
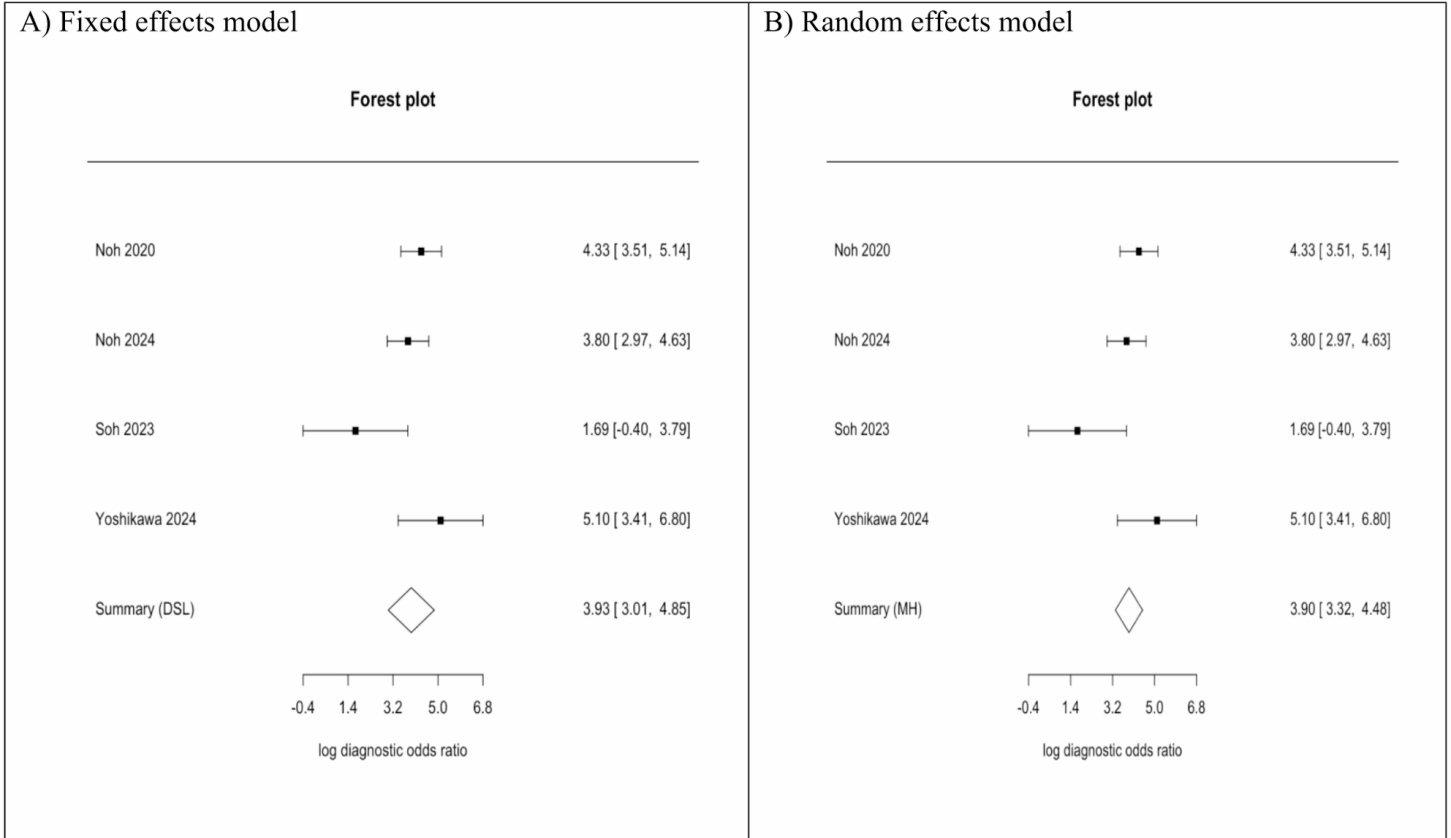
Forest plots for (**A**) pooled diagnostic odds ratios using the fixed effects model, (**B**) pooled diagnostic odds ratios using the random effects model

Two models were built to pool the effect sizes. The DOR of the fixed effects model was 3.90 [ 3.32, 4.48], while the random effects model was 3.93 [ 3.01, 4.85], Fig. 3.

#### Confusion matrix

**Fig. 4:**
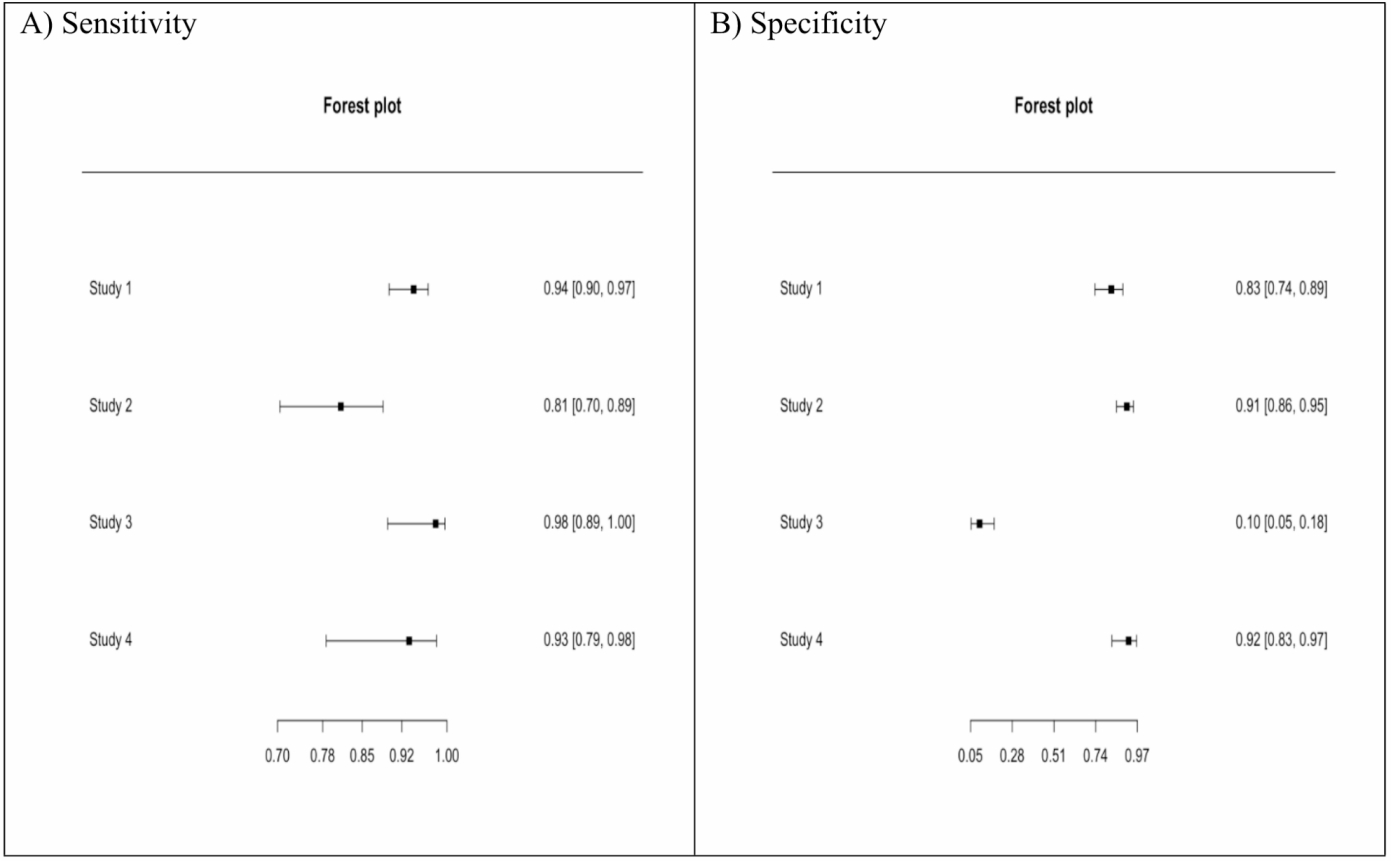
Forest plots for (**A**) sensitivity, (**B**) specificity

**Fig. 5:**
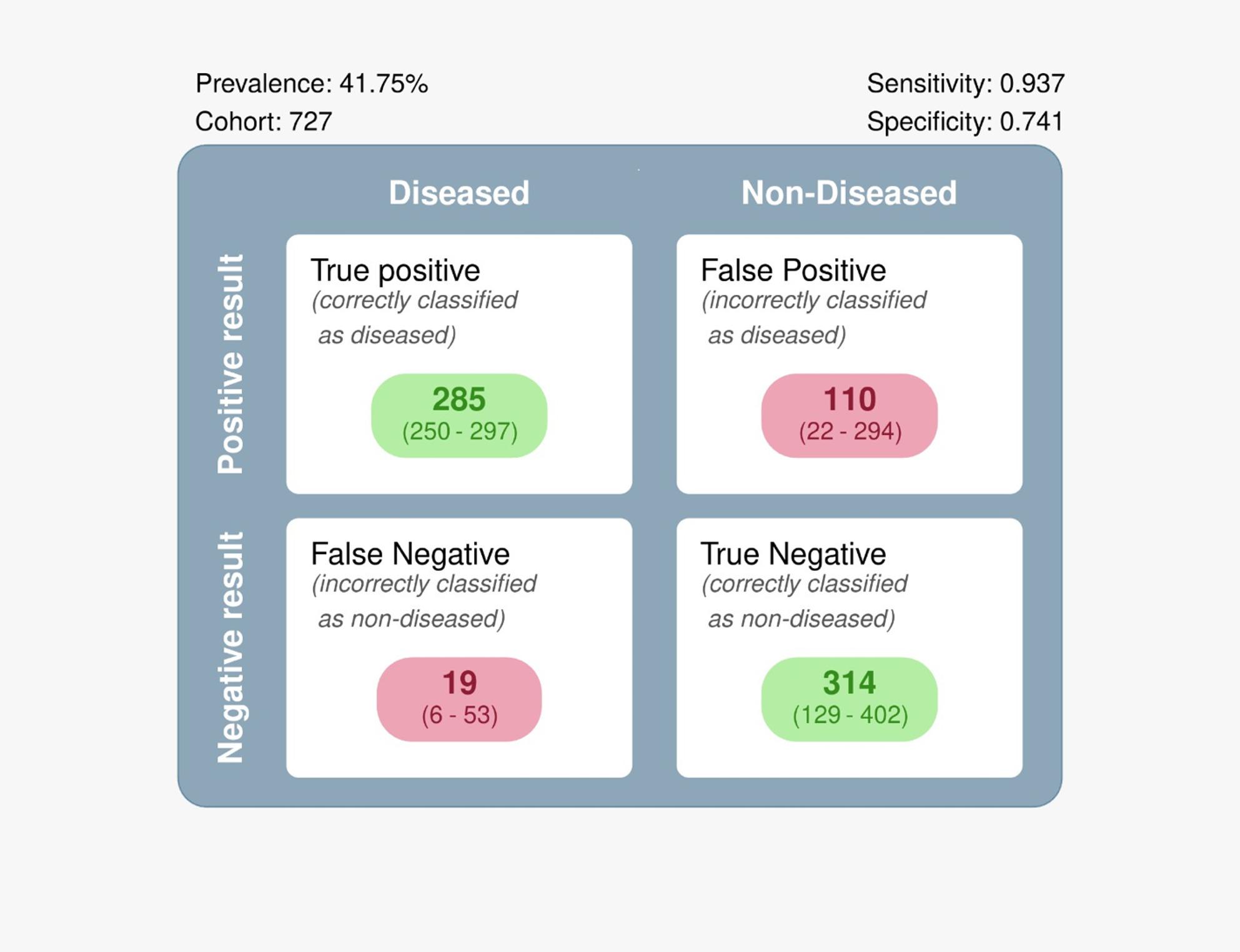
Confusion matrix

A confusion matrix was used to summarize the previously mentioned results. Gastric swabs reported a high sensitivity of 93.7%, with a high number of true positives, TP = 285. However, test specificity is moderate (74.1%). False positives (110) generally outnumber false negatives (19); however, false negatives are much more critical in a clinical setting as they represent missed cases. A wide confidence interval in both true negatives (129–402) and false positives (22–294) confirms the previously mentioned results of a higher heterogeneity between the included studies in terms of test specificity, as shown in Figs. 4 and 5.

## Discussion

*H. Pylori* was first identified in dogs’ stomachs in 1892 [20]. *H. Pylori* is the most common bacterial infection, affecting more than half of the world’s population. It has been reported that *H. Pylori* incidence increases with age during the first 10 years of life. Higher incidence rates of childhood *H. Pylori* are reported in underdeveloped countries, reaching 90%. The exact cause of the infection is still a mystery. However, multiple contributing factors have been suggested, including environmental factors and low socioeconomic status [21].

*H. Pylori* transmission methods are still unclear. The suggested transmission routes are oral-oral, fecal-oral, and gastro-oral, either directly from one person to another or indirectly through the surrounding environment [20, 21]*H. Pylori* is a gram-negative bacillus that survives in the gastric mucosa, causing a plethora of gastric manifestations, including peptic ulcer disease (PUD), gastric adenocarcinoma, and gastric mucosa-associated lymphoid tissue (MALT) lymphoma [22]. Some *H. pylori-*related extra-gastric diseases have been reported, including unexplained iron-deficiency anemia, vitamin B12 deficiency, and idiopathic thrombocytopenic purpura. A relationship between *H. Pylori* and asthma and allergy has been reported in the USA. However, it has not been confirmed. *H. Pylori* is an infectious disease; its treatment is pivotal for the well-being of society. The first line of treatment is triple proton pump inhibitors (PPIs) in all patients. However, antibiotic resistance remains a stubborn stone in the PPIs’ pathway. Most commonly, resistance occurs against clarithromycin and metronidazole. Multiple solutions are used to counteract this problem, including applying a hybrid therapy of PPIs plus three antibiotics [23].

This is the first meta-analysis to compare gastric swabs and biopsies and urease breath tests. Our results reported that gastric swab has a high sensitivity of 93.7%, with a high number of true positives of 285, across the four included studies, Noh et al. [19], Noh et al. [16], Soh et al., and Yoshikawa et al. However, test specificity was moderate (74.1%), with a wide confidence interval, which can be attributed to the smaller sample size of the Soh et al. study. The results showed that false positives (110) were more than false negatives (19). However, false negatives are more clinically critical as they represent those with the disease who have not been diagnosed. These results were maintained even with variability between the included studies. Our meta-analysis supports using gastric swabs as an alternative for diagnosing *H. Pylori* with some considerations. In our meta-analysis, the pooled Diagnostic Odds Ratio was highly consistent across models. The fixed-effects model yielded a DOR of 3.90 (95% CI: 3.32–4.48), while the random-effects model produced a very similar estimate of 3.93 (95% CI: 3.01–4.85). The close agreement between the two approaches suggests that the overall discriminative ability of the test is robust and not materially influenced by between-study variability. The wider confidence interval under the random-effects model reflects expected heterogeneity, but the central effect size remains stable.

Due to the high prevalence rates of *H. Pylori*, multiple different diagnostic methods have been proposed and studied for rapid and accurate diagnosis. Diagnostic measures are divided into two main types: invasive and non-invasive. Invasive methods include endoscopy, histopathology, and rapid urease test (RUT). Non-invasive techniques include the urea breath test (UBT), stool antigen test (SAT), and serology. Molecular testing, like polymerase chain reaction (PCR), can be both invasive and non-invasive, according to the case [24].

Due to the huge burden *H. Pylori* infection puts on the world’s population, multiple studies have been dedicated to comparing diagnostic methods with the aim of finding the optimal tool.

A comparative study by AbuTaleb et al. in 2018 reported a sensitivity of 100% and 59% specificity in RUT results compared to other diagnostic methods, including non-invasive ones. The study concluded that invasive studies are much more accurate, with non-invasive ones being best used in follow-up [25]. Novel endoscopic diagnostic procedures, such as magnifying blue laser imaging endoscopy and high-definition magnifying endoscopy, were found to have high sensitivity and positive predictive value [26].

A meta-analysis reporting the efficacy of non-invasive tools reported high sensitivity and specificity of UBT (96.4%, 88.3%) and helicobacter stool antigen (HpSA) (72.5%, 94.7%). On the other hand, serology had high sensitivity rates of 83.7%, while specificity rates were low (73.3%). These offer an alternative for elderly patients, especially those recently undergoing an endoscopy (Omar, 2023).

The strength of this study lies in following PRISMA guidelines and using reliable tools for risk of bias assessment and accurate conduct of analysis. However, this study is still limited to available literature and the low sample size used in some of the included studies. Additionally, three studies were based in Korea, while two of them were from the same group, Noh 2020 and Noh 2024. Further testing and investigation should be done on a larger scale with appropriate safety precautions.

## Conclusion

This systematic review compared gastric swab-based RUT and biopsy or UBT for *H. pylori* detection. It showed high sensitivity (93.7%) for detecting *H. pylori*, suggesting its potential to identify most cases of infection reliably. However, the moderate specificity (74.1%) showed that there is some risk of false positives. These results indicated that gastric swab-based RUT may be helpful as an initial diagnostic test, specifically if a biopsy is difficult. More studies with large sample sizes are needed to confirm these findings.

## Supplementary Information

Supplementary Material 1.

## Data Availability

This published article and its supplementary materials include all data generated or analyzed during this study.
