# Supplementary material for: Efficacy of swab-based RUT in detecting H. pylori infection: a systematic review and meta-analysis
Source: BMC Gastroenterol. 2026 Mar 27;26:214. doi: 10.1186/s12876-025-04576-6 (PMC13064230; doi:10.1186/s12876-025-04576-6)
Supplement: Supplementary file 1 — Supplementary Material 1. [file 12876_2025_4576_MOESM1_ESM.docx]

| Database | Search Terms | Search Field | Search Results |
| --- | --- | --- | --- |
| PubMed | (Sweeping OR swab OR sweep OR swabbing) AND ("Helicobacter pylori" OR "H. pylori" OR "Campylobacter pylori" OR "Campylobacter pyloridis" OR "Helicobacter nemestrinae") | All Field | 58 |
| WOS | (Sweeping OR swab OR sweep OR swabbing) AND ("Helicobacter pylori" OR "H. pylori" OR "Campylobacter pylori" OR "Campylobacter pyloridis" OR "Helicobacter nemestrinae") | All Field | 85 |
| SCOPUS | (Sweeping OR swab OR sweep OR swabbing) AND ("Helicobacter pylori" OR "H. pylori" OR "Campylobacter pylori" OR "Campylobacter pyloridis" OR "Helicobacter nemestrinae") | Title, Abstract, Keywords | 92 |
| Cochrane | (Sweeping OR swab OR sweep OR swabbing) AND ("Helicobacter pylori" OR "H. pylori" OR "Campylobacter pylori" OR "Campylobacter pyloridis" OR "Helicobacter nemestrinae") | Title, Abstract, Keywords | 6 |

Table S I: Search terms and results in different databases.


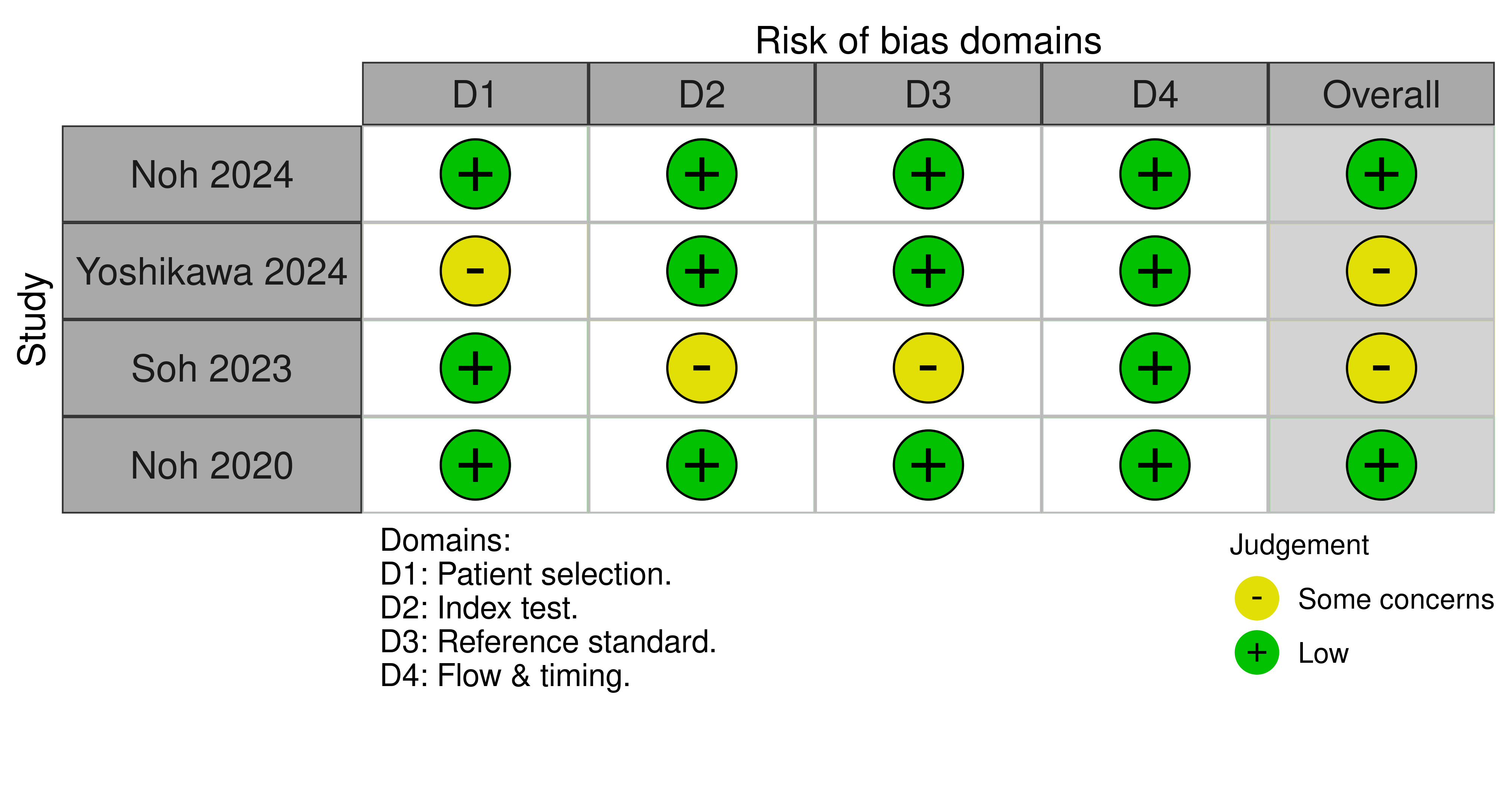


Figure S1: QUADAS-2 for the included studies.
